# Myostatin Knockout Regulates Bile Acid Metabolism by Promoting Bile Acid Synthesis in Cattle

**DOI:** 10.3390/ani12020205

**Published:** 2022-01-15

**Authors:** Di Wu, Mingjuan Gu, Zhuying Wei, Chunling Bai, Guanghua Su, Xuefei Liu, Yuefang Zhao, Lei Yang, Guangpeng Li

**Affiliations:** State Key Laboratory of Reproductive Regulation and Breeding of Grassland Livestock, College of Life Science, Inner Mongolia University, Hohhot 010021, China; wudi2020imu@163.com (D.W.); gmj0119@yeah.net (M.G.); weizhuying2008@126.com (Z.W.); chunling1980_0@163.com (C.B.); suguanghua0707@163.com (G.S.); liuxuefei1006@126.com (X.L.); nklana@163.com (Y.Z.)

**Keywords:** myostatin, serum, liver, metabolomics, bile acid

## Abstract

**Simple Summary:**

Myostatin (MSTN) gene knockout can increase lean muscle mass and has been widely used in livestock breeding. MSTN deficiency also regulates various metabolic processes. However, the effect of MSTN knockout on the liver, the largest metabolic organ, has not been reported. In this study, physiological and biochemical parameters of serum, untargeted and targeted metabolomics of MSTN^+/−^ and WT cattle were studied, and we found that the knockout of MSTN could regulate liver metabolism and promote bile acid metabolism. This may be due to the enhanced expression of bile acid synthesis genes in MSTN^+/−^ bovine livers. In conclusion, MSTN knockout regulated bile acid metabolism via enhanced bile acid synthesis.

**Abstract:**

Myostatin (MSTN) is a major negative regulator of skeletal muscle mass and causes a variety of metabolic changes. However, the effect of MSTN knockout on bile acid metabolism has rarely been reported. In this study, the physiological and biochemical alterations of serum in MSTN^+/−^ and wild type (WT) cattle were investigated. There were no significant changes in liver and kidney biochemical indexes. However, compared with the WT cattle, lactate dehydrogenase, total bile acid (TBA), cholesterol, and high-density lipoprotein (HDL) in the MSTN^+/−^ cattle were significantly increased, and glucose, low-density lipoprotein (LDL), and triglycerides (TG) were significantly decreased, indicating that MSTN knockout affected glucose and lipid metabolism and total bile acids content. Targeted metabolomic analysis of the bile acids and their derivatives was performed on serum samples and found that bile acids were significantly increased in the MSTN^+/−^ cattle compared with the WT cattle. As the only bile acid synthesis organ in the body, we performed metabolomic analysis on the liver to study the effect of MSTN knockout on hepatic metabolism. Metabolic pathway enrichment analysis of differential metabolites showed significant enrichment of the primary bile acid biosynthesis and bile secretion pathway in the MSTN^+/−^ cattle. Targeted metabolomics data further showed that MSTN knockout significantly increased bile acid content in the liver, which may have resulted from enhanced bile acid synthesis due to the expression of bile acid synthesis genes, cholesterol 7 alpha-hydroxylase (CYP7A1) and sterol 27-hydroxylase (CYP27A1), and upregulation in the liver of the MSTN^+/−^ cattle. These results indicate that MSTN knockout does not adversely affect bovine fitness but regulates bile acid metabolism via enhanced bile acid synthesis. This further suggests a role of MSTN in regulating metabolism.

## 1. Introduction

Myostatin (MSTN) belongs to the transforming growth factor beta (TGFβ) superfamily. It is the primary negative regulator of skeletal muscle growth and development [1]. Inactivation of MSTN by engineered deletion or natural mutation leads to muscle hypertrophy in mice, cattle, humans, sheep, dogs, pigs, and rabbits [2,3,4,5,6,7]. MSTN has critical roles in many aspects of metabolism such as glucose and lipid metabolism [8,9]. Hormone levels in vivo are key to glucose metabolism, such as insulin and thyroid hormones [10,11,12]. MSTN inactivation attenuates skeletal muscle insulin resistance and regulates glucose metabolism [8]. Meanwhile, MSTN deficiency has an effect on lipids, which can promote brown fat formation in white adipose tissue and fatty acid oxidation to prevent obesity under a high fat diet [9]. It has also been reported that MSTN dysfunction decreased the cholesterol of plasma in mice under caloric restriction [13].

The liver is the largest regulating organ of glucose and lipid metabolism in the body and is the main site of gluconeogenesis, cholesterol production and metabolism, lipoprotein uptake and secretion, fat production and fatty acid β-oxidation [14,15,16]. The digested monosaccharides in the small intestine are absorbed through the mucous membrane to the liver where glycogen is synthesized and stored. When blood glucoses are consumed in large quantities, liver glycogen can be broken down into glucoses and enter circulation [17]. The absorbed fatty acids enter the liver and are converted into functional fatty acids such as cholesterol, phospholipids, and bile acids; the liver is the center of fatty acids transport [18]. The liver is also the only site of cholesterol metabolization into bile acids [19]. Bile acids exist in the body not only by themselves, but also in a variety of derivatives, which enter the circulation system to maintain the homeostasis of the body [20]. Recently, the role of bile acids in the integrated regulation of lipid, glucose, and energy metabolism has been identified [21]. Bile acids have been shown to mediate multiple signals to regulate energy homeostasis. For example, bile acids modulate glucose metabolism via both FXR and TGR5 and regulate lipid metabolism by GPCR and S1P2 [22,23]. However, there is no report on the effect of MSTN knockout on hepatic metabolism.

In this study, we found that MSTN knockout upregulated cholesterol and bile acid content in serum. To further investigate the underlying causes, we performed a comprehensive profiling of metabolism in the liver based on untargeted metabolomics, which showed that MSTN knockout regulated bile acid metabolism in the liver. Targeted metabolomics and the detection of bile acid synthesis gene expression demonstrated that MSTN knockout regulated bile acid metabolism probably via upregulating hepatic bile acid biosynthesis.

## 2. Materials and Methods

### 2.1. Animals and Sample Collection

As in our previous report [24], we used CRISPR/Cas9 and somatic cell nuclear transfer to generate MSTN knockout Luxi cattle. In this study, the MSTN^+/−^ cattle used were a cross between wild type female Luxi cattle and male MSTN^−/−^ Luxi cattle. A total of 20 cattle, 10 MSTN^+/−^ Luxi cattle (5 female and 5 male) and 10 wild type Luxi cattle (5 female and 5 male) were used. Female cattle calving parities were 0, not in lactation. The wild type cattle had not undergone gene editing nor been crossbred and were pure Luxi breed. The cattle were fed in Hohhot, China (111°85′ E, 40°55′ N, 1040 m above sea level). Each barn contained about 240 m^2^ of indoor space and 300 m^2^ of exercise yard, which can keep 15~20 cattle. Female and male cattle were kept in separate cowsheds in the same environment, and each animal could move freely indoors and outdoors without restraint. Each cowshed was equipped with a constant temperature (15 °C) automatic watering system, and all cattle were free to drink water. The total mixed ration (TMR) diet consisted of 70% silage, 10% hay/alfalfa, and 20% supplementary grain feed. The forage/concentrate ratio was 4:1. The supplemental grain feed (Inner Mongolia Meng Yuan Kang Feed Co., Ltd. Hohhot, China) contained maize, soybean meal, soy flour, DDGS (Distillers Dried Grains with Solubles), calcium carbonate, calcium hydrogen phosphate, sodium chloride, trace elements, and vitamins. On the day of blood collection, the cattle had empty stomachs, and 10 mL of blood was collected from their jugular veins. The blood was slowly poured into a 15 mL centrifuge tube and centrifuged at 3000 r for 10 min. The prepared serum was transferred into 1.5 mL cryo-storage tubes, labeled, and stored in liquid nitrogen. The cattle were slaughtered at the age of 24 months and fasted for 24 h before slaughter. Slaughter started in the morning, and all cattle were slaughtered by exsanguination. The process of slaughter followed the national standard operating procedures (GB/T 19477-2018, Cattle Slaughtering, China). The livers were collected within 30 min after slaughter and cut into several pieces and placed quickly in liquid nitrogen, followed by storage at −80 °C until further use.

### 2.2. Serum Physiological and Biochemical Analyses

The serum physiological and biochemical parameters were detected by a Cobas C 311 analyzer (Roche, Mannheim, Germany, 68305), and data collection was accomplished by the built-in software (software version 01–09) as described [25]. The parameters of low-density lipoprotein (LDL) (Roche, Germany, 07005717190), glucose (Roche, Germany, 04404483190), lactate (Roche, Germany, 03183700190), lactate dehydrogenase (LDH) (Roche, Germany, 03004732122), α-amylase (Roche, Germany, 03183742122), total bile acid (TBA) (Roche, Germany, 03333825190), cholesterol (Roche, Germany, 03039773190), lipase (Roche, Germany, 03029590322), total protein (Roche, Germany, 03183734190), albumin (Roche, Germany, 03183688122), alanine aminotransferase (ALT) (Roche, Germany, 04467388190), high-density lipoprotein (HDL) (Roche, Germany, 07528566190), aspartate aminotransferase (AST) (Roche, Germany, 20764949322), cholinesterase (Roche, Germany, 04498631190), triglycerides (TG) (Roche, Germany, 20767107322), creatinine (Roche, Germany, 04810716190), and urea (Roche, Germany, 04460715190) were measured and analyzed. Non-esterified fatty acids (NEFA) and β-hydroxybutyrate (BHB) were assayed using a non-esterified fatty acids assay kit (Jiancheng, China, A042-1-1) and β-Hydroxybutyrate assay kit (Jiancheng, China, E030-1-1). Receiver operating characteristic (ROC) curves were performed using the OmicStudio tools at https://www.omicstudio.cn/tool/58, accessed on 31 October 2021.

### 2.3. Western Blot

The total protein was extracted from the liver of the MSTN^+/−^ cattle by homogenization in ice-cold radio immunoprecipitation assay (RIPA) buffer. The liver tissue lysates were then centrifuged at 4 °C for 30 min at 8000× *g*. The protein concentration was determined by BCA assay (Thermo, Waltham, MA, USA, 23225). The supernatant was electrophoresed in 8–12% SDS-polyacrylamide gel and transferred onto a polyvinylidene difluoride membrane by electroblotting. The membrane was blocked in 5% non-fat milk in Tris-buffered saline with 0.1% Tween-20 (TBST) blocking solution at room temperature for 1 h, and incubated with anti-CYP7A1 (Abcam, Cambridge, MA, USA, ab234982) and anti-CYP27A1 (Abcam, Cambridge, MA, USA, ab227248) in TBST containing 0.5% non-fat milk at 4 °C overnight. The membranes were then incubated for 1 h with a horseradish peroxidase-conjugated goat anti-mouse and anti-rabbit secondary antibodies (1:10,000) at room temperature, followed by detection using the chemiluminescence labeling detection reagent ECL Plus (Thermo, Waltham, MA, USA, 32209). *GAPDH* was used as the loading control. Original Western blot images could be found at Figure S1.

### 2.4. Real-Time PCR

The RNA from the liver was extracted using an RNAiso Plus kit (Takara, Shiga, Japan, 9108) following the manufacturer’s protocol. cDNA was synthesized by a PrimeScript RT reagent kit with gDNA Eraser (Perfect Real Time) (Takara, Shiga, Japan, RR047A). We amplified the cDNA using ABI7500 real-time PCR (Applied Biosystems, Foster City, CA, USA) and SYBR Green (Takara, Shiga, Japan, RR820A). The following primers were used: *CYP7A1*: forward 5′-TCGAACTGGAGCTTGTGGAGAGC-3′, reverse 5′-CGCAGCCTTGTAACAGCACCAG-3′; *CYP27A1*: forward 5′-CTGCTGCTGACAAGGCTGATCC-3′, reverse 5′-CCAGAACGATGCGAGCCACAC-3′; *GAPDH*: forward 5′-GTGGCAAAGTGGAGATTGTTG-3′, reverse 5′-CTCCTGGAAGATGGTGATGG-3′. The protocol for PCR amplifications was as follows: 95 °C for 30 s, followed by 40 cycles at 95 °C for 5 s, 60 °C for 34 s, and a final melting curve stage. For normalization, the housekeeping gene *GAPDH* was used as the endogenous control and fold-changes in gene expression were determined using the comparative threshold cycle (2^−∆∆Ct^) method.

### 2.5. Targeted Metabolomics Analysis of Bile Acids in Liver and Serum 

After thawing the serum samples and homogenizing the liver samples, methanol containing 67% water was added and centrifuged at 4 °C for 14,000× *g* for 10 min to remove proteins. The supernatant was dried under nitrogen flow, and then redissolved using 100 µL in a 50% aqueous methanol solution containing 0.005% formic acid for UPLC-MS/MS analysis. All samples were injected into the Phenomenex Kinetex C18 (2.1 × 100 mm, 2.6 µm) at a flow rate of 0.35 mL/min. The mobile phase consisted of 0.005% formic acid in water (A) and 0.005% formic acid in chromatographic pure acetonitrile (B). Agilent Masshunter Analyst software was used for instrument control and data acquisition.

### 2.6. Metabolomic Analysis of Liver 

Three milliliters of methanol and 0.64 mL water were added to each gram of tissue samples and homogenized in an ice bath. The samples were centrifuged at 4 °C, 15,000× *g* for 10 min, the lower organic layers (with lipophilic compounds) were transferred into separate vials for LC-MS (LC-Bio, Hangzhou, China) analysis. Metabolite separations were performed with ACQUITY UPLC HSS T3 column (100 mm × 2.1 mm, 1.8 m, Waters, Manchester, UK) to analyze the liver tissue samples. The mass spectrometer was operated in both positive and negative ion mode for the analysis. XCMS [26], CAMERA [27], and the metaX [28] toolbox were used to convert raw data files. Metabolites were identified using KEGG and HMDB [29] (http://www.hmdb.ca/, accessed on 11 October 2021) metabolic databases. The potential metabolites were screened based on the variable importance in the projection (VIP) values and Student’s *t*-test. VIP  >  1 and *p*  <  0.05 were considered as statistically significant. Enrichment analysis on the differentially expressed genes was carried out by Kyoto Encyclopedia of Genes and Genomes (KEGG) (https://www.omicshare.com/tools/, accessed on 11 October 2021).

### 2.7. Statistical Analysis

The data were presented as the mean ± SD of three independent experiments. Statistical analyses were performed using the GraphPad Prism 8.3.0 software. Student’s *t*-tests were used to calculate the *p*-values: *p* < 0.05 was considered statistically significant difference.

## 3. Results

### 3.1. Analysis of Serum Physiological and Biochemical Parameters 

**Figure 1:**
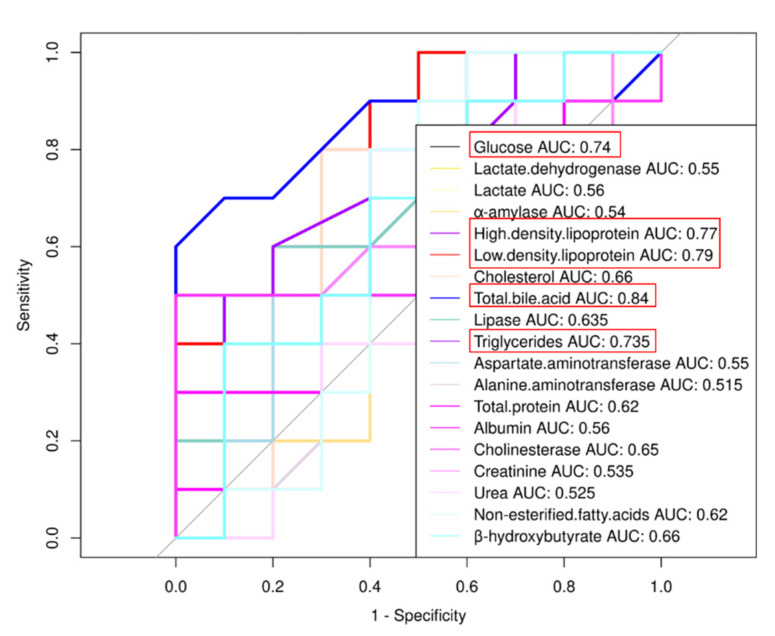
Receiver operating characteristic (ROC) curve analysis of biochemical indexes. If the area under the curve (AUC) is greater than 0.70, parameters are considered correlation.

According to the physiological and biochemical indexes in the serum (Table 1), the glucose level of the MSTN^+/−^ cattle was significantly lower than that of the WT cattle (*p* = 0.0183), and the lactate dehydrogenase amount was significantly higher (*p* = 0.0333). No significant differences were observed in α-amylase (*p* = 0.229) and lactate contents (*p* = 0.5878). Lipid metabolism test results showed that total bile acid (TBA) (*p* = 0.00001), cholesterol (*p* = 0.0011), and high-density lipoprotein (HDL) (*p* = 0.0014) were significantly increased in the MSTN^+/−^ cattle in comparison with the WT cattle. Low-density lipoprotein (LDL) (*p* = 0.0028) and triglycerides (TG) (*p* = 0.0227) were significantly lower in the MSTN^+/−^ cattle than the WT cattle. There was no difference in the lipase amount (*p* = 0.6375) between the MSTN^+/−^ and the WT cattle. The total protein (*p* = 0.1232), albumin (*p* = 0.9857), alanine aminotransferase (ALT) (*p* = 0.5942), aspartate aminotransferase (AST) (*p* = 0.4076), and cholinesterase (*p* = 0.1704) in the MSTN^+/−^ cattle did not show any significant difference from the WT cattle. The renal biomarkers of creatinine (*p* = 0.3795) and urea (*p* = 0.0874) showed no difference between the MSTN^+/−^ and the WT cattle. In addition, the levels of non-esterified fatty acids (NEFA) (*p* = 0.8454) and β-hydroxybutyrate (BHB) (*p* = 0.7876), two important parameters of bovine metabolism [30,31], were measured. The results showed no difference between the two groups (Table 1). Receiver operating characteristic (ROC) curve analysis was carried out for biochemical indexes. If the area under the curve (AUC) was greater than 0.70, there parameters were considered correlation. As shown in Figure 1, the expression of MSTN was strongly correlated with glucose, HDL, LDL, TBA, and TG. These results indicated that there were no significant differences in the liver and kidney functions between the MSTN^+/−^ and the WT cattle, while there were significant changes in the serum glucose metabolism, lipid metabolism, and total bile acid levels. Thus, MSTN knockout could regulate the metabolism of glucose and lipids in cattle. 

### 3.2. MSTN Knockout Altered Bile Acid Metabolism in Serum

**Figure 2:**
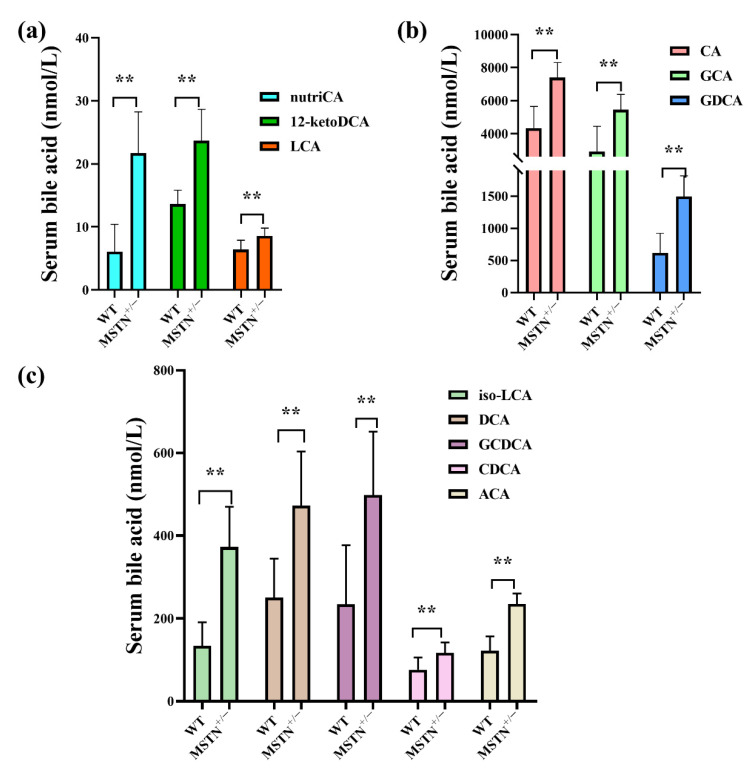
Targeted metabolomics analysis identifies the alteration of bile acids in serum of MSTN^+/−^ and WT cattle. (**a**–**c**) Differentially expressed bile acid species were identified. ** *p* < 0.01. WT, wild type; MSTN^+/−^, MSTN heterozygous deletion. Abbreviations: nutriCA, nutritional cholic acid; 12-ketoDCA, 12-ketodeoxycholic acid; CA, cholic acid; GCA, glycol-cholic acid; GDCA, glycodeoxycholic acid; iso-LCA, isolithocholic acid; DCA, deoxycholic acid; GCDCA, glycol-chenodeoxycholic acid; LCA, lithocholic acid; CDCA, chenodeoxycholic acid; ACA, acetylcholic acid.

To determine the effect of MSTN knockout on bile acid homeostasis, we measured 19 bile acids in serum using targeted metabolomics. Compared with the WT cattle, the MSTN^+/−^ cattle had significantly increased the contents of cholic acid (CA), glycol-cholic acids (GCA), glycodeoxycholic acid (GDCA), isolithocholic acid (iso-LCA), deoxycholic acid (DCA), glycol-chenodeoxycholic acids (GCDCA), chenodeoxycholic acids (CDCA), acetylcholic acid (ACA), nutritional cholic acid (nutriCA), 12-ketodeoxycholic acid (12-ketoDCA), and lithocholic acid (LCA) (Figure 2a–c). These results suggested that MSTN knockout significantly increased serum bile acid content in the MSTN^+/−^ cattle, indicating that it affected the serum bile acid metabolism.

### 3.3. Effect of MSTN Knockout on Metabolism of Liver

**Figure 3:**
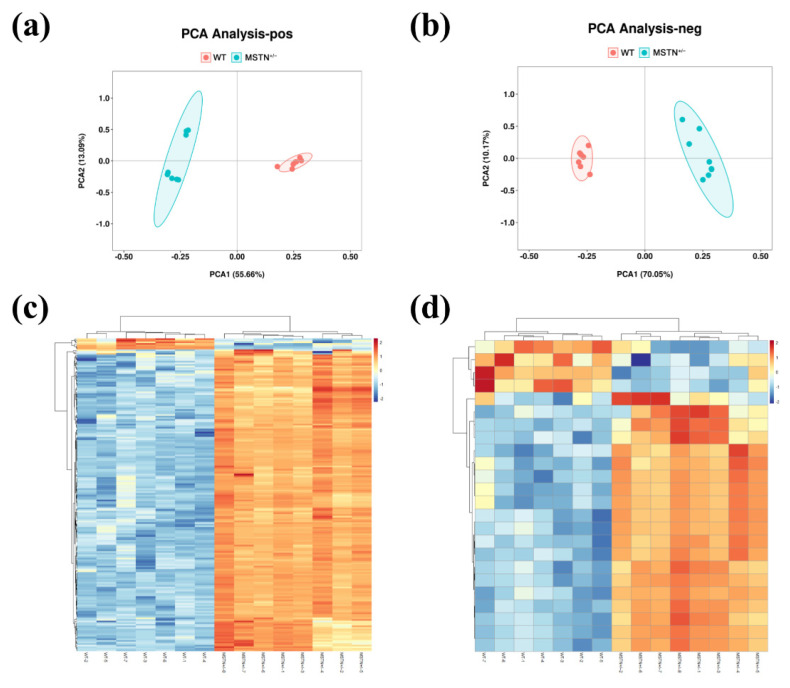
Untargeted metabolomics analysis of liver in MSTN^+/−^ and WT cattle. Principal component analysis (PCA) of metabolic data in positive (**a**) and negative (**b**) ion mode. Heat map of differential metabolites in positive (**c**) and negative (**d**) ion mode. WT, wild type; MSTN^+/−^, MSTN heterozygous deletion.

Since liver is the only organ capable of synthesizing bile acids, we assessed whether MSTN knockout had an effect on hepatic metabolism. In the principal component analysis (PCA), untargeted metabolomics showed a clear separation between the MSTN^+/−^ and WT cattle (Figure 3a,b). The differential metabolites were identified by KEGG, HMDB Database and metaX software. In positive ion mode, a total of 442 differential metabolites were found in the MSTN^+/−^ cattle compared with the WT cattle. In the MSTN^+/−^ cattle, 394 differential metabolites were upregulated and 48 were downregulated (Figure 3c). In negative ion mode, a total of 95 metabolites were differentially expressed in the MSTN^+/−^ cattle compared with the WT cattle. In the MSTN^+/−^ cattle, 83 differential metabolites were elevated and 12 were decreased (Figure 3d). KEGG enrichment analysis showed that, in the positive ion mode, the differential metabolites were mainly enriched in primary bile acid biosynthesis (map00120), fatty acid degradation (map00071), taurine and hypotaurine metabolism (map00430), alpha-linolenic acid metabolism (map00592), bile secretion (map04976), and drug metabolism-cytochrome p450 (map00982) (Table 2). In the negative ion mode, the differential metabolites were mainly enriched in primary bile acid biosynthesis, bile secretion, neomycin, kanamycin and gentamicin biosynthesis, and taurine and hypotaurine metabolism (Table 2). The metabolomic analysis indicated that MSTN knockout could significantly affect liver metabolism, especially bile acid metabolism.

### 3.4. MSTN Knockout Promoted Bile Acids Metabolism in Liver

**Figure 4:**
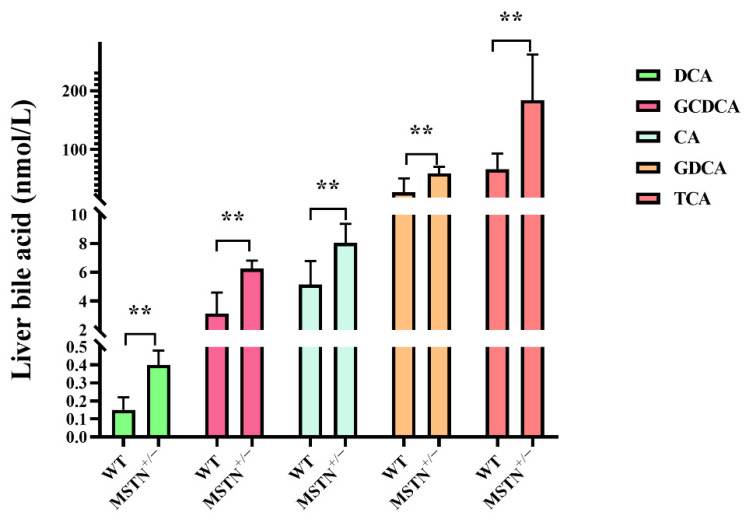
Targeted metabolomics analysis identifies the alteration of bile acid in the liver of MSTN^+/−^ and WT cattle. Differentially expressed bile acid species were identified in the liver. ** *p* < 0.01. WT, wild type; MSTN^+/−^, MSTN heterozygous deletion. Abbreviations: DCA, deoxycholic acid; GCDCA, glycol-chenodeoxycholic acid; CA, cholic acid; GDCA, glycodeoxycholic acid; TCA, taurocholic acid.

In order to further confirm liver untargeted metabolomics findings, metabolomic profiles of hepatic bile acids were examined by absolute quantification of the targeted metabolomic. Compared with the WT cattle, the contents of deoxycholic acid (DCA), glycol-chenodeoxycholic acids (GCDCA), cholic acid (CA), glycodeoxycholic acid (GDCA) and taurocholic acid (TCA) in the MSTN^+/−^ cattle were significantly upregulated. There were no significant differences in the contents of tauro-alphamuricholic acid (T-α-MCA), taurochenodeoxycholic acid (TCDCA), glycol-cholic acids (GCA), glycoursodeoxycholic acid (GUDCA), glycolythocholic acid (GLCA), acetylcholic acid (ACA), chenodeoxycholic acids (CDCA), taurodeoxycholic acid (TDCA), ursodeoxycholic acid (UDCA), nutritional cholic acid (nutriCA), 12-ketodeoxycholic acid (12-ketoDCA), taurolithocholic acid (TLCA), lithocholic acid (LCA), and isolithocholic acid (iso-LCA) between the MSTN^+/−^ and WT cattle (Figure 4). These results further indicated that MSTN knockout promotes hepatic bile acid metabolism and leads to increased hepatic bile acid contents.

### 3.5. MSTN Knockout Upregulated Expression of Genes Related to Bile Acid Synthesis

**Figure 5:**
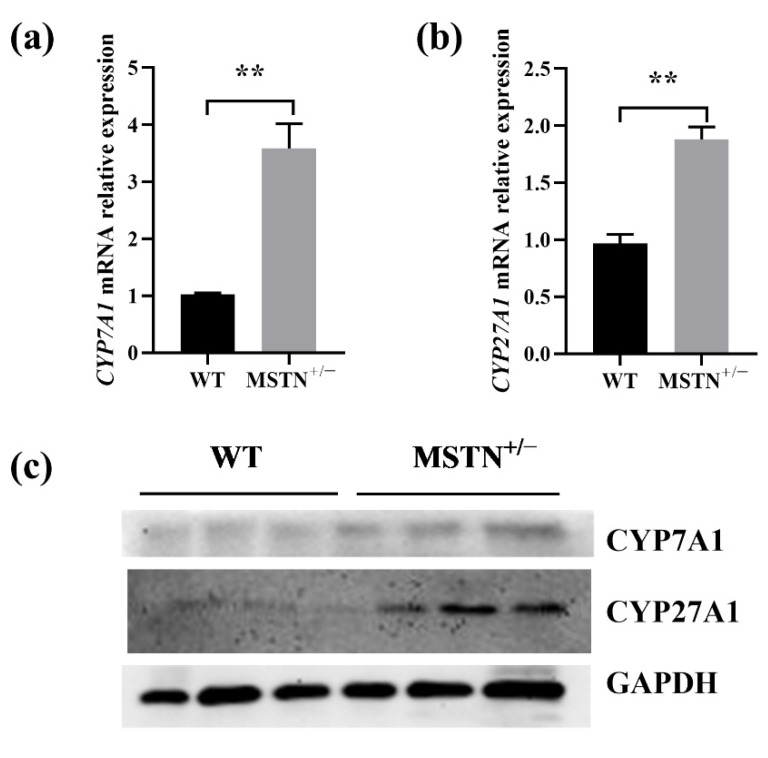
MSTN knockout increased expression of genes related to bile acid synthesis in the liver of MSTN^+/−^ and WT cattle. (**a**,**b**) Expression of cholesterol 7 alpha-hydroxylase (CYP7A1) and sterol 27-hydroxylase (CYP27A1) mRNA. (**c**) Expression of CYP7A1 and CYP27A1 protein. ** *p* < 0.01. WT, wild type; MSTN^+/−^, MSTN heterozygous deletion.

Since the liver is the major site of bile acid synthesis, we asked whether the MSTN^+/−^ cattle regulated bile acid synthesis. Two genes encoding the key enzymes for the classical and alternative pathway of bile acid synthesis were upregulated in the MSTN^+/−^ cattle (Figure 5). As in Figure 5a,b, the mRNA levels of cholesterol 7 alpha-hydroxylase (CYP7A1) and sterol 27-hydroxylase (CYP27A1) in the MSTN^+/−^ cattle were significantly increased. Consistent with the mRNA results, the protein levels of CYP7A1 and CYP27A1 were significantly upregulated in the MSTN^+/−^ cattle compared with the WT cattle (Figure 5c). These results suggest that the MSTN^+/−^ cattle lead to increased content of hepatic bile acids by upregulating the expression of genes associated with bile acid synthesis.

## 4. Discussion

Myostatin is a well-known negative regulator of skeletal muscle mass [32]. Currently, a number of natural mutations in the myostatin gene have been found in cattle breeding, resulting in a 20% increase in total muscle mass [33]. In developed muscle with MSTN gene deletion, the lipolysis signal was downregulated and oxidative metabolism was weakened [34]. In addition, deletion of the MSTN gene significantly reduced lipid storage, mobilization, and oxidation [35]. The downregulation of lipid metabolism caused by MSTN gene deletion was balanced by increased glucose oxidation [36]. Guo et al. found increased glucose utilization, uptake, and insulin sensitivity in muscle and adipose tissue of MSTN knockout mice [36]. In MSTN knockout muscle, glucose uptake markers were significantly increased [37]. In addition, glucose transporter 4 (GLUT4), responsible for glucose transport, was found to be regulated by MSTN in Japanese shorthorn cattle [38]. Fatty acids and glucose are known to be the main energy sources of skeletal muscle; MSTN may help coordinate the use of these two substrates [35]. In our previous reports, we have shown that the MSTN^−/+^ cattle derived from CRISP/Cas9-edited bull and non-edited cows significantly changed glycolysis, glycogen metabolism, and fatty acid β-oxidation [39,40]. Serum physiological and biochemical parameters can reflect the physiological and metabolic status of the entire body in animals [41]. In the present study, the glucose level of the MSTN^+/−^ cattle was significantly lower than that of the WT cattle, and lactate dehydrogenase was significantly higher. Lipid metabolism test results showed that total bile acid (TBA), cholesterol, and the high-density lipoprotein (HDL) were significantly increased in the MSTN^+/−^ cattle compared with the WT cattle. Low-density lipoprotein (LDL) and triglycerides (TG) were significantly lower in the MSTN^+/−^ cattle than the WT cattle. However, total protein, albumin, alanine aminotransferase (ALT), aspartate aminotransferase (AST), and cholinesterase in the MSTN^+/−^ cattle did not show any significant difference from the WT cattle. The renal biomarkers of creatinine and urea also showed no difference between the MSTN^+/−^ and WT cattle. These results confirmed that both liver and renal function have no significant difference between the MSTN^+/−^ and WT cattle, but glucose metabolism, lipid metabolism, and the content of total bile acids in serum changed significantly.

The liver is the largest internal organ and controls organism metabolism [42]. Myostatin inhibits proliferation and insulin-stimulated glucose uptake in liver cells [43]. MSTN deficiency reduced liver weight in MSTN knockout piglets [44], mitigated sepsis-induced liver dysfunction [45], and protected the liver against obesity-induced insulin resistance [46]. In this study, we found that MSTN knockout changed bile acid metabolic-related pathways, such as primary bile acid biosynthesis and bile secretion in the liver.

Bile acid is synthesized in liver cells and is the end product of cholesterol metabolism. Bile acids enter the gallbladder through canaliculi to form bile. They are released into the duodenum after stimulation to emulsify and decompose lipids. Then, bile acids are circulated back to the hepatocytes via the circulatory system [47]. The bile acids are synthesized by the liver (called primary bile acids), enter the intestine and are further modified by bacteria in the cecum and colon into secondary bile acids [48,49]. The bacteria involved in the processing of secondary bile acids, including Firmicutes, Bacteroidetes, and Actinobacteria [50]. Bacteria of the *Clostridium*, *Bacteroides*, *Lactobacillus*, *Bifidobacterium*, and *Enterococcus* genus have been proved to be involved in the production of secondary bile acids [51]. In this study, the concentrations of 19 primary and conjugated bile acids in the liver were significantly increased in the MSTN^+/−^ cattle, and the secondary bile acids were also dramatically increased in the serum. Elevated liver and serum bile acid contents imply that there was a change of bile acid metabolism in the MSTN^+/−^ cattle. Furthermore, MSTN knockout changed the composition of the metabolites and microbial strains in the jejunum and cecum of MSTN^+/−^ large white pigs [52]. In future work, we will analyze the microbes and metabolites in the gut of MSTN^+/−^ cattle to provide more details about bile acids.

Bile acids are synthesized from cholesterol in the liver via two main pathways: the classical and alternative pathways [53,54]. There are two ways, that is, classical and alternative pathways, to synthesize bile acids in the liver to replenish the body’s bile acid pool. The classical pathway is the main pathway of bile acid synthesis, and CYP7A1 is the primary rate-limiting enzyme in this pathway [55,56]. The alternative bile acid pathway in the liver is initiated by CYP27A1 when CYP7A1 is not expressed [57]. In this study, to elucidate the mechanism of the changes of bile acid induced by MSTN knockout, the gene expression associated with bile acid synthesis was analyzed by quantitative real-time PCR and Western blot. Protein and mRNA levels of both CYP7A1 and CYP27A1 were significantly increased in the liver of the MSTN^+/−^ cattle. These results suggest that MSTN knockout may promote bile acid synthesis by upregulating the expression of CYP7A1 and CYP27A1, but it still needs to be verified.

## 5. Conclusions

MSTN knockout did not cause changes in the serum physiological indexes, but increased bile acid content in both the liver and serum, which may upregulate the expressions of CYP7A1 and CYP27A1 in the liver of MSTN^+/−^ cattle.

## Figures and Tables

**Table 1:** Serum physiological and biochemical parameters of MSTN^+/^^−^ and WT cattle.

Items	WT	MSTN^+/−^	*p*-Value
Glucose (mmol/L)	4.5 ± 0.92	3.65 ± 0.51 *	0.0183
Lactate dehydrogenase (U/L)	1038.45 ± 332.6	1301 ± 147.62 *	0.0333
Lactate (mmol/L)	1.66 ± 0.74	1.49 ± 0.57	0.5878
α-amylase (U/L)	25.78 ± 13.23	32.08 ± 9.46	0.229
High-density lipoprotein (mmol/L)	1.81 ± 0.63	3.07 ± 0.34 **	0.0014
Low-density lipoprotein (mmol/L)	1.16 ± 0.28	0.69 ± 0.34 **	0.0028
Cholesterol (mmol/L)	2.02 ± 0.74	3.07 ± 0.48 **	0.0011
Total bile acid (mmol/L)	12.37 ± 2.2	18.15 ± 2.07 **	0.00001
Lipase (U/L)	13.18 ± 4.83	12.36 ± 2.58	0.6375
Triglycerides (mmol/L)	0.32 ± 0.07	0.23 ± 0.07 *	0.0227
Aspartate aminotransferase (U/L)	50.06 ± 14.68	55.55 ± 13.38	0.4076
Alanine aminotransferase (U/L)	21.61 ± 5.84	23.23 ± 6.49	0.5942
Total protein (g/L)	45.67 ± 13.46	56.46 ± 13.49	0.1232
Albumin (g/L)	30.44 ± 7.5	30.38 ± 6.5	0.9857
Cholinesterase (U/L)	129.64 ± 39.64	149.4 ± 19.52	0.1704
Creatinine (umol/L)	125.2 ± 41.98	139 ± 19.27	0.3795
Urea(mmol/L)	3.23 ± 1.05	2.46 ± 0.8	0.0874
Non-esterified fatty acids (mmol/L)	0.297 ± 0.096	0.304 ± 0.117	0.8454
β-hydroxybutyrate (mmol/L)	0.408 ± 0.116	0.397 ± 0.129	0.7876

* *p* < 0.05, ** *p* < 0.01. WT, wild type; MSTN^+/−^, MSTN heterozygous deletion.

**Table 2:** Enrichment of metabolic pathways based on differentially expressed metabolites of liver in MSTN^+/^^−^ and WT cattle.

Pathway	Pathway ID	*p*-Value
neg-WT_MSTN^+/−^		
Primary bile acid biosynthesis	map00120	0.000629646
Bile secretion	map04976	0.00383948
Neomycin, kanamycin, and gentamicin biosynthesis	map00524	0.0233545
Taurine and hypotaurine metabolism	map00430	0.03357927
pos-WT_MSTN^+/−^		
Primary bile acid biosynthesis	map00120	0.000644895
Fatty acid degradation	map00071	0.008414756
Taurine and hypotaurine metabolism	map00430	0.008414756
Alpha-linolenic acid metabolism	map00592	0.009727567
Bile secretion	map04976	0.02492269
Drug metabolism-cytochrome p450	map00982	0.02970364

WT, wild type; MSTN^+/−^, MSTN heterozygous deletion.

## Data Availability

The data presented in this study are available on request from the corresponding author.

## Supplementary Materials

The following supporting information can be downloaded at: https://www.mdpi.com/article/10.3390/ani12020205/s1, Figure S1: Full original blots used for Figure 5c, each blot membrane was cut based on the standard band positions and then incubated with the appropriate antibodies. The bands in the article are marked in a red frame, and the bands not included in the article are marked in green frame.
