# Supplementary material for: Myostatin Knockout Regulates Bile Acid Metabolism by Promoting Bile Acid Synthesis in Cattle
Source: Animals (Basel). 2022 Jan 15;12(2):205. doi: 10.3390/ani12020205 (PMC8772948; doi:10.3390/ani12020205)
Supplement: Supplementary file 1 [file animals-12-00205-s001.zip › animals-1498271-supplementary.pdf]

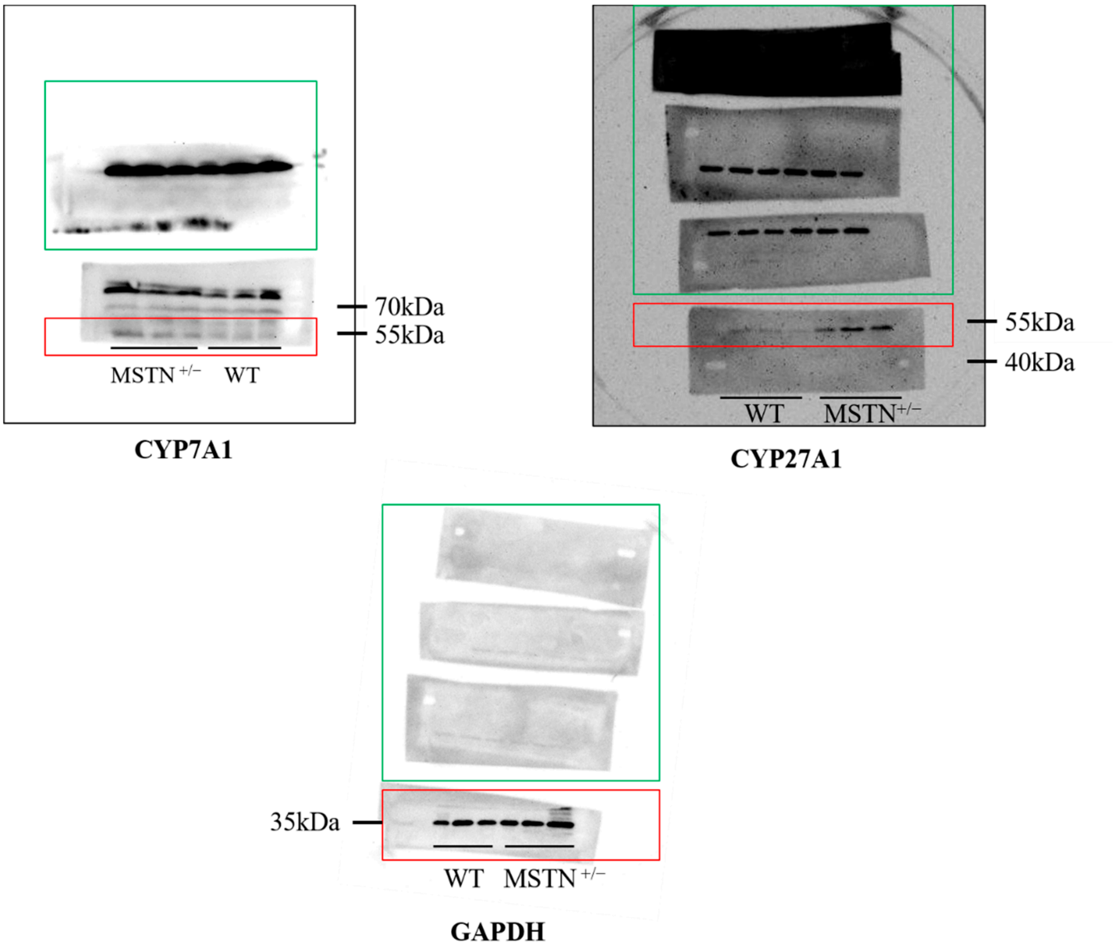

Figure S1. Full original blots used for Figure 5c, each blot membrane was cut based on the standard band positions and then incubated with the appropriate antibodies. The bands in the article are marked in a red frame, and the bands not included in the article are marked in green frame.
